# Effect of levosimendan on prognosis in adult patients undergoing cardiac surgery: a meta-analysis of randomized controlled trials

**DOI:** 10.1186/s13054-017-1848-1

**Published:** 2017-10-17

**Authors:** Qi-Hong Chen, Rui-Qiang Zheng, Hua Lin, Jun Shao, Jiang-quan Yu, Hua-Ling Wang

**Affiliations:** 1 Department of Critical Care Medicine, Subei People’s Hospital, School of Medicine, Yangzhou University, 98 Nantong West Road, Yangzhou, Jiangsu 225001 People’s Republic of China; 2 Department of Cardiology, Subei People’s Hospital, School of Medicine, Yangzhou University, 98 Nantong West Road, Yangzhou, 225001 People’s Republic of China

**Keywords:** Levosimendan, Cardiac surgery, Mortality, Meta-analyses

## Abstract

**Background:**

Small trials suggest that levosimendan is associated with a favorable outcome in patients undergoing cardiac surgery. However, recently published larger-scale trials did not provide evidence for a similar benefit from levosimendan. We performed a meta-analysis to assess the survival benefits of levosimendan in patients undergoing cardiac surgery and to investigate its effects in subgroups of patients with preoperative low-ejection fraction (EF).

**Methods:**

We identified randomized clinical trials through 20 April 2017 that investigated levosimendan therapy versus control in patients undergoing cardiac surgery. Individual patient data from each study were compiled. Meta-analyses were performed for primary outcomes, secondary outcomes and serious adverse events, and subgroup analyses according to the preoperative EF of enrolled patients were also conducted. The risk of bias was assessed using the Cochrane risk-of-bias tool.

**Results:**

Seventeen studies involving a total of 2756 patients were included. Levosimendan therapy was associated with a significant reduction in 30-day mortality (RR 0.67; 95% CI, 0.49 to 0.93; *p* = 0.02) and reduced the risk of death in single-center trials (RR 0.49; 95% CI, 0.30 to 0.79; *p* = 0.004) and in subgroup trials of inferior quality (RR 0.39; 95% CI, 0.17 to 0.92; *p* = 0.02); however, in multicenter and in high-quality subgroup-analysis trials, no significant difference in mortality was observed between patients who received levosimendan therapy and controls (*p* > 0.05). However, in high-quality subgroup trials, levosimendan therapy was associated with reduced mortality in patients in a preoperative low-EF subgroup (RR 0.58; 95% CI, 0.38 to 0.88; p = 0.01). Similarly, only patients in the preoperative low-EF subgroup benefited in terms of reduced risk of renal replacement therapy (RR 0.54; 95% CI, 0.34 to 0.85; *p* = 0.007). Furthermore, levosimendan therapy was associated with a significant reduction in intensive care unit (ICU) length of stay (MDR −17.19; 95% CI, −34.43 to −2.94; *p* = 0.02).

**Conclusions:**

In patients undergoing cardiac surgery, the benefit of levosimendan in terms of survival was not shown in multicenter or in high-quality trials; however, levosimendan therapy was associated with reduced mortality in patients with preoperative ventricular systolic dysfunction.

**Electronic supplementary material:**

The online version of this article (doi:10.1186/s13054-017-1848-1) contains supplementary material, which is available to authorized users.

## Background

Many patients with advanced stages of cardiac disease need cardiac surgery, which can lead to severe left ventricular dysfunction and, in particular, postoperative low cardiac output syndrome (LCOS) [1]. These conditions may lead to increased morbidity, multiple organ failure and death [2]. Strategies to address the syndrome include the use of inotropic agents and an intra-aortic balloon pump (IABP). Unfortunately, most inotropic agents increase postoperative morbidity and mortality rates due to increased myocardial oxygen consumption [3].

Levosimendan is an inotropic agent that enhances myocardial contractility without increasing myocardial oxygen demand in patients with low cardiac systolic dysfunction [4]. Clinical studies have reported that levosimendan therapy improves survival in patients undergoing cardiac surgery [5, 6]. Previous meta-analyses of small randomized trials have shown that levosimendan is associated with survival benefits among patients undergoing cardiac surgery [7, 8]. However, recently published larger-scale trials did not provide evidence for a similar benefit from levosimendan [9–11].

The objective of this meta-analysis was to assess the survival benefits of levosimendan in patients undergoing cardiac surgery. Previous meta-analyses showed that patients with low ejection fraction (EF) benefit more from levosimendan therapy than patients with normal EF [7, 8]. Therefore, we performed subgroup analyses according to the preoperative EF of enrolled patients.

## Methods

### Eligibility criteria

We included trials with the following features:

1. Type of study: randomized controlled clinical trials
2. Population: patients undergoing cardiac surgery
3. Intervention: patients receiving intravenous levosimendan
4. The following outcomes were included
  - Primary outcomes: 30-day mortality or in-hospital mortality.
  - Secondary outcomes: requiring renal replacement therapy, the duration of mechanical ventilation, and intensive care unit (ICU) length of stay.
  - Serious adverse events: the occurrence of postoperative arrhythmia and hypotension.

### Search strategy for the identification of studies

We conducted a search of the Medline, Elsevier, Cochrane (Central), Web of Science and ClinicalTrials.gov databases for studies investigating the perioperative use of levosimendan in patients undergoing cardiac surgery. When searching each database, the term “levosimendan” was combined with the Cochrane highly sensitive search strategy for identifying randomized trials [12]. Searches were limited to English and included all publications in the available databases through 20 April 2017.

### Study selection

Two reviewers independently screened abstracts and titles to determine whether the studies met the inclusion criteria. The full texts of the articles were then reviewed independently in accordance with the inclusion and exclusion criteria. Studies that reported randomized clinical trials of the preoperative or postoperative administration of levosimendan in patients undergoing cardiac surgery were included. “low-EF studies” were defined as studies that limited their analysis to patients with a preoperative EF ≦40% or enrolled patients with a preoperative mean EF ≦40%. The remaining studies were designated “preserved-EF studies” [13]. Any discrepancies were resolved by reaching a consensus on the inclusion or exclusion of a study by discussion with a third reviewer.

### Data extraction and management

Two authors independently extracted the data using a standardized data extraction protocol. Any disagreements between the two reviewers were resolved through discussion, after which consensus was reached. Some means and standard deviations of the patients’ duration of mechanical ventilation and ICU length of stay data were estimated according to the method described by Hozo [14]. Information including trial characteristics, criteria for inclusion and exclusion, the method of intervention and outcomes was extracted from the included studies.

### Methodological quality assessment

Two reviewers independently completed a risk of bias assessment following the instructions of the Cochrane Collaboration tool for systematic reviews of interventions [12]. The items are defined as sequence generation; allocation concealment; blinding; incomplete outcome reporting; and other risks of bias. Each item included in the Cochrane Collaboration tool was reported in terms of unclear, low or high risk of bias. Disagreements were resolved via discussion, and a third reviewer mediated situations where disagreements occurred.

### Trial sequential analysis

We perform a trial sequential analysis (TSA) to prevent the risk of random error from being increased by repeated updates. We used TSA-adjusted random-effects modes to pool results from the included studies for primary outcomes. A one-sided TSA was conducted to maintain a 5% risk of type I error and 80% power. Furthermore, we used the estimated function to calculate the required information size.

### Statistical analysis

For the meta-analysis, data from the included studies were analyzed using Review Manager (Review Manager, version 5.3), and pooled risk ratios for dichotomous data and mean differences for continuous data with 95% CIs were calculated. The statistical heterogeneity of the data was quantified using the Mantel-Haenszel chi-square test and the *I* ^2^ test. Any obvious heterogeneity was predefined as *p* < 0.05 using the Mantel-Haenszel chi-square test or *I* ^2^ > 50%. Furthermore, publication bias was assessed using funnel plot techniques.

## Results

### Study location and selection

**Fig. 1:**
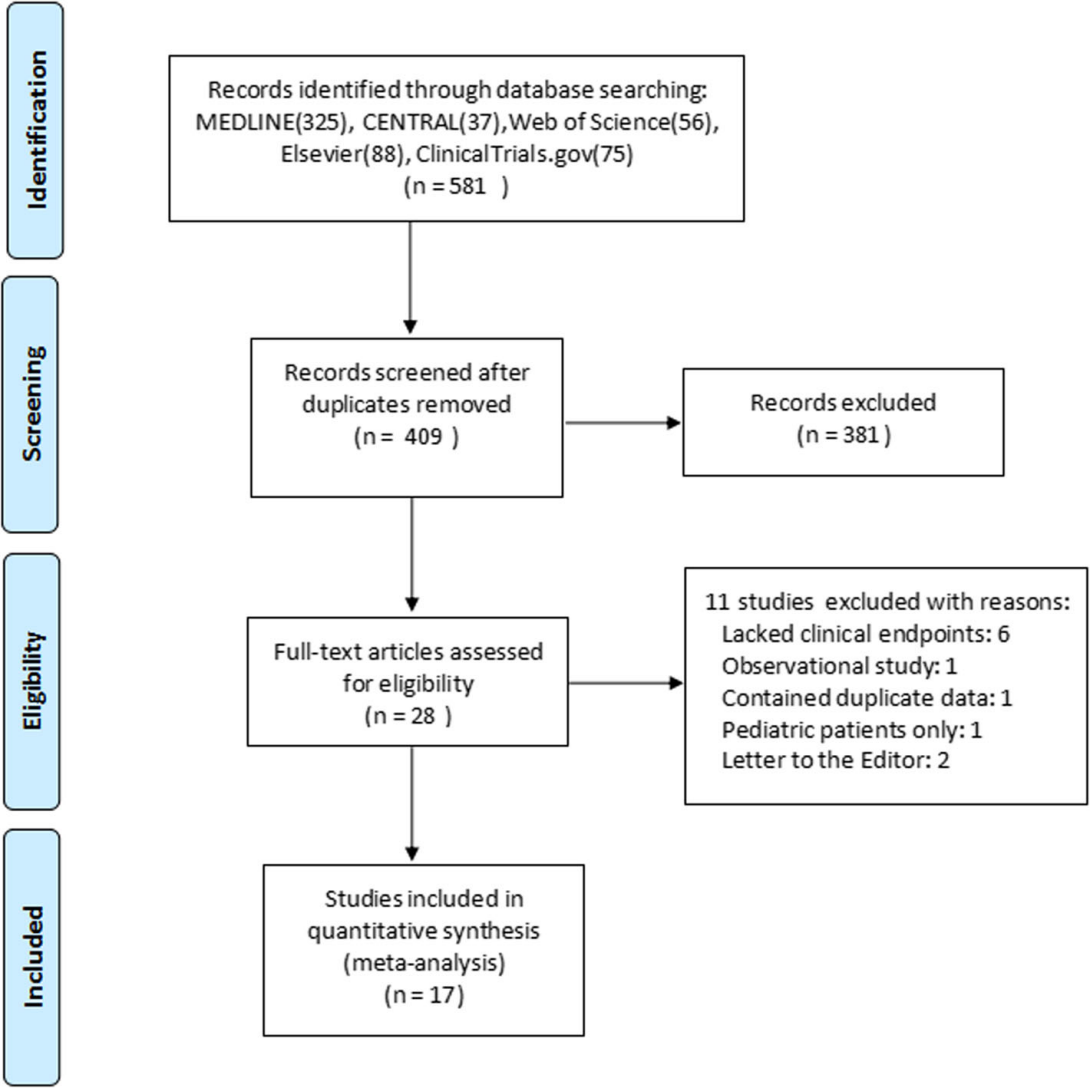
Flow diagram of the identified trials

We identified a total of 581 titles and abstracts after the primary search. After screening the abstracts, 553 articles were found to be repeated or non-relevant and were therefore excluded. The remaining 28 articles were retrieved for an eligibility assessment, as the result of which 11 studies were deemed ineligible and were therefore excluded. Seventeen studies with a total of 2756 patients were included in the final analysis (Fig. 1).

### Characteristics of the trials

We included sixteen trials that compared levosimendan with controls in patients undergoing cardiac surgery. The characteristics of the included studies are shown in Table 1. Four multicenter trials [9–11, 15] and 12 single-center trials were included [5, 6, 16–26]. Five studies [9, 18–20, 23] including patients with preoperative mean EF > 40% were assigned to the preserved-EF subgroup. Lahtinen 2011 [18] did not report mean or median EF but did report that most patients had an EF > 50%. Thus, this study was assigned to the preserved-EF subgroup. The remaining 12 studies [5, 6, 11, 15–17, 21, 22, 24–26] were categorized as the low-EF subgroup as they involved patients with preoperative mean EF ≤ 40%.

**Table 1:** Characteristics of included studies

				EF			
Study	N	Centre/country	Setting	Levosimendan	Control	Levosimendan dosage	Control
Landoni 2017	506	Multicenter/Italy	CABG or HVR	50 (37–59)	50 (40–60)	Bolus: none Inf: 0.025 to 0.2 ug/kg/min Duration: 48 h	Placebo
Mehta 2017	849	Multicenter/USA	CABG or HVR	26 (24–32)	27 (22–31)	Bolus: 0.2 ug/kg/min. Inf: 0.1 ug/kg/min Duration: 24 h	Placebo
Cholley 2017	335	Multicenter/French	CABG or HVR	<40%	< 40%	Bolus: none Inf: 0.1ug/kg/min Duration: 24 h	Placebo
Anastasiadis 2016	32	Single/Greece	CABG or HVR	35.7 ± 4.9	37.5 ± 3.4	Bolus: none Inf: 0.1ug/kg/min Duration: 24 h	Placebo
Baysal 2014	128	Single/Turkey	HVR	35.0 (20–45)	35 (25–45)	Bolus: 6ug/kg Inf: 0.1 ug/kg/min Duration: 24 h	Standard therapy
Erb 2014	33	Single/Germany	CABG or HVR	22.0 ± 4.5	22.4 ± 5.5	Bolus: none Inf: 0.1 ug/kg/min Duration:24 h	Placebo
Levin 2012	252	Single/USA	CABG	< 25%	< 25%	Bolus:10ug/kg Inf: 0.1 ug/kg/min Duration:24 h	Placebo
Lomivorotov 2012	60	Single/Russia	CABG	28.8 ± 4.0	27.8 ± 5.4	Bolus:12ug/kg Inf: 0.1 to 0.2 ug/kg/min Duration: 24 h	IABP
Lahtinen 2011	200	Single/Finland	CABG or HVR	More than 50%	More than 50%	Bolus: 24ug/kg Inf: 0.2 ug/kg/min Duration: 24 h	Placebo
Tritapepe 2009	102	Single/Italy	CABG	41.6 ± 10.7	44.1 ± 9.8	Bolus: 24 ug/kg Duration: 10 min	Placebo
De Hert 2007	30	Single/Belgium	CABG	24 ± 6	27 ± 3	Bolus: none Inf: 0.1 ug/kg/min Duration: 24 h	Placebo
Tritapepe 2006	24	Single/Italy	CABG	50 ± 7	52 ± 5	Bolus: 24 ug/kg Duration: 10 min	Placebo
Al-Shawaf 2006	30	Single/Kuwait	CABG	29 ± 6	31 ± 6	Bolus: 12ug/kg Inf: 0.1 ug/kg/min Duration: 24 h	Placebo
Eriksson 2009	60	Multicenter/Finland	CABG	36 ± 8	36 ± 8	Bolus: 12 ug/kg Inf: 0.2 ug/kg/min Duration: 24 h	Placebo
Leppikangas 2011	24	Single/Finland	CABG or HVR	63 ± 9	69 ± 9	Bolus: 12 ug/kg Inf: 0.2 ug/kg/min Duration: 24 h	Placebo
Alvarez 2006	41	Single/Spain	CABG or HVR	35.5 ± 4.2	33.2 ± 5.2	Bolus: 12ug/kg Inf: 0.2 ug/kg/min Duration: 24 h	Dobutamine
Shah 2014	50	Single/India	CABG	22.5 ± 4.1	22.6 ± 3.4	Bolus: none Inf: 0.13 ug/kg/min Duration: 24 h	Placebo

*CABG* coronary artery bypass grafting, *HVR* heart valve replacement, *EF* ejection fraction, *IABP* intra-aortic balloon counterpulsation, *Inf* infusion, *USA* United States of America

### Bias risk assessment

**Fig. 2:**
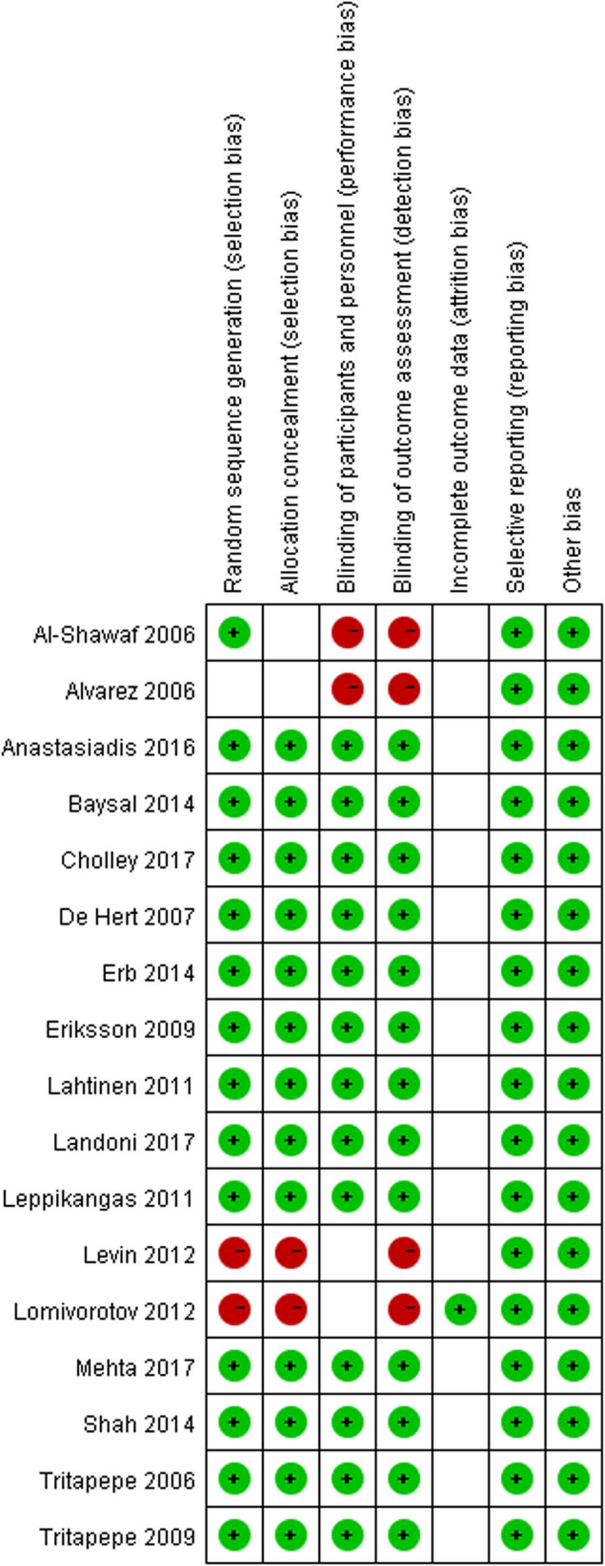
Risk of bias summary. Review of authors’ judgments about each risk-of-bias item for each included study. Red, high risk; green, low risk; blank, unclear

Random sequence generation was assessed as a low risk of bias in 14 studies (82%), allocation concealment was assessed in 12 studies (75%), blinding of participants was assessed in 13 studies (76%), blinding of outcome assessors was assessed in 13 studies (76%), incomplete outcome data was assessed in one study (6%) and selective outcome reporting was assessed in all studies (100%). Thirteen trials [5, 6, 9–11, 15, 18–21, 23, 25, 26] with low risk of bias were assigned to the high-quality trials subgroup, while the remaining four trials [16, 17, 22, 24] with moderate or high risk of bias were assigned to the inferior quality subgroup (Fig. 2).

### TSA

**Fig. 3:**
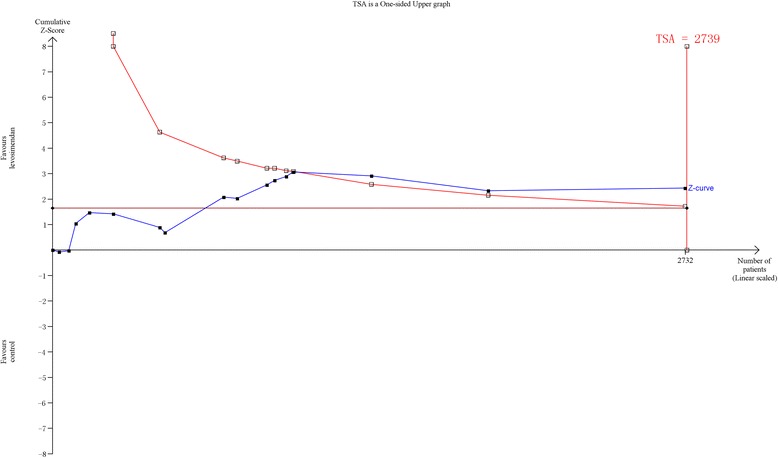
Trial sequential analysis (TSA) for mortality in randomized controlled trials: one-sided boundary, incidence of 8.59% in the control arm, incidence of 5.99% in the treatment arm, low-bias estimated relative-risk reduction of 80%, α of 5%, and power of 80% were set. There is an estimated required information sample size of 2739 randomized patients, which was not achieved. The boundaries for futility are crossed

A sensitivity analysis of TSA including all trials revealed that the diversity-adjusted information size was 2739 patients. The cumulative z-curve crossed the conventional boundary for benefit and the trial sequential monitoring boundary for benefit but did not cross the estimated information size boundary (Fig. 3). The TSA evaluations suggested that this meta-analysis could draw firm conclusions although the data were insufficient.

### Mortality

**Fig. 4:**
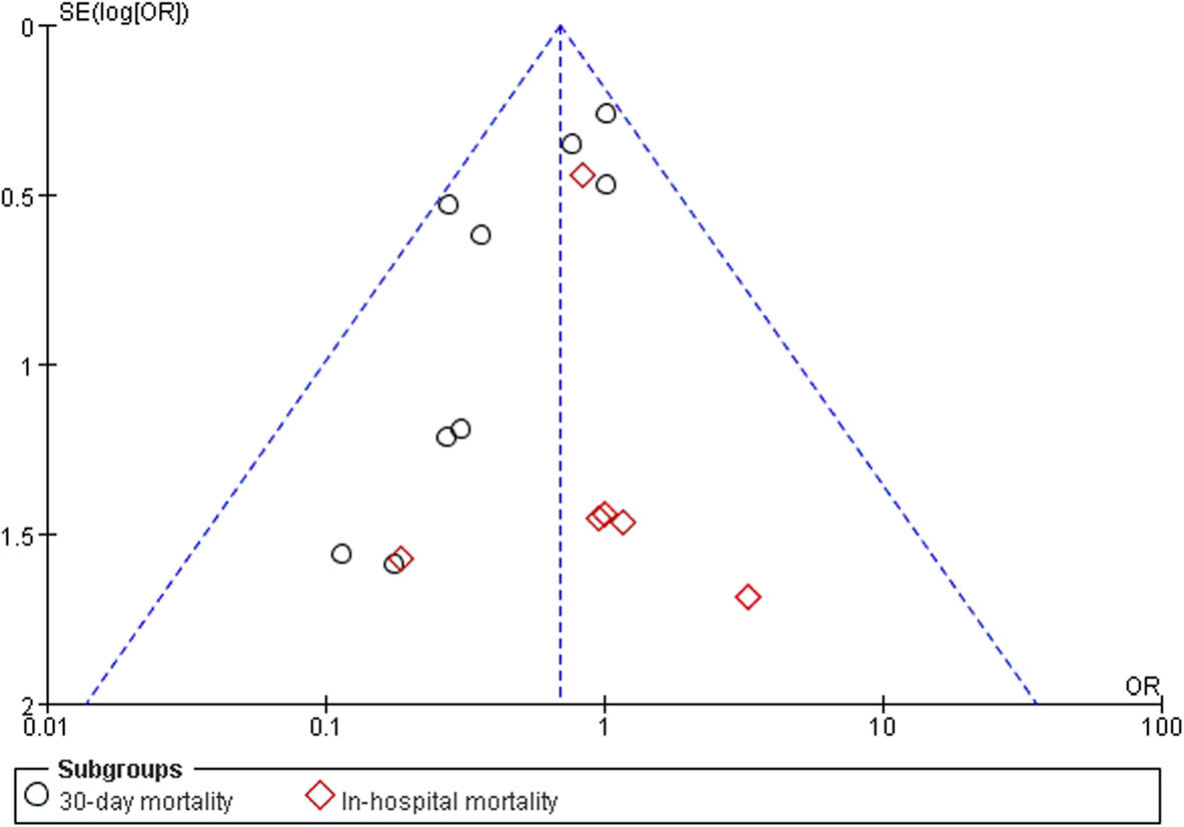
The funnel plot for mortality demonstrates there is no publication bias

**Fig. 5:**
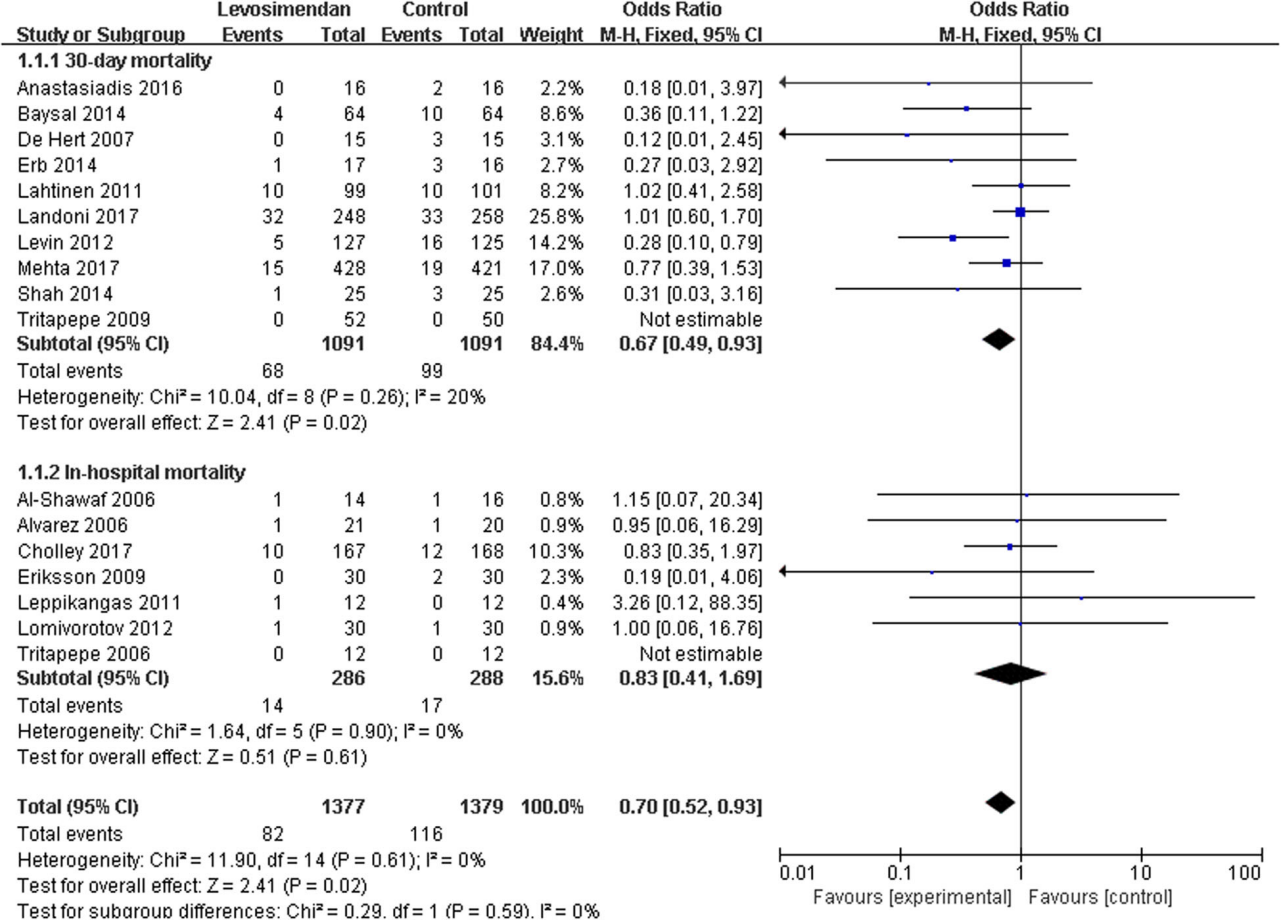
The effect of levosimendan therapy on postoperative mortality in patients undergoing cardiac surgery. M-H, Mantel-Haenszel

The effect of levosimendan on mortality rates was estimated from 17 trials and included a total of 2756 patients. Thirty-day mortality was reported for 10 studies [5, 6, 9, 10, 16, 18, 20, 21, 25, 26] and in-hospital mortality was reported for the remaining 7 studies [11, 15, 17, 19, 22–24]. A total of 82 deaths occurred among 1377 patients allocated to the levosimendan group compared with 116 deaths among 1379 patients allocated to the control group. We detected no evidence of publication bias following funnel plot analysis (Fig. 4), and heterogeneity was determined to be non-significant (*p* = 0.61, *I* ^2^ = 0). The result showed a significant reduction in the overall risk of death following the levosimendan intervention (RR 0.70; 95% CI, 0.52 to 0.93; *p* = 0.02). Levosimendan significantly reduced 30-day mortality (RR 0.67; 95% CI, 0.49 to 0.93; *p* = 0.02) but not in-hospital mortality (RR 0.83; 95% CI, 0.41 to 1.69; *p* = 0.61, Fig. 5).

### Subgroup analyses of single-center or multicenter trials

**Fig. 6:**
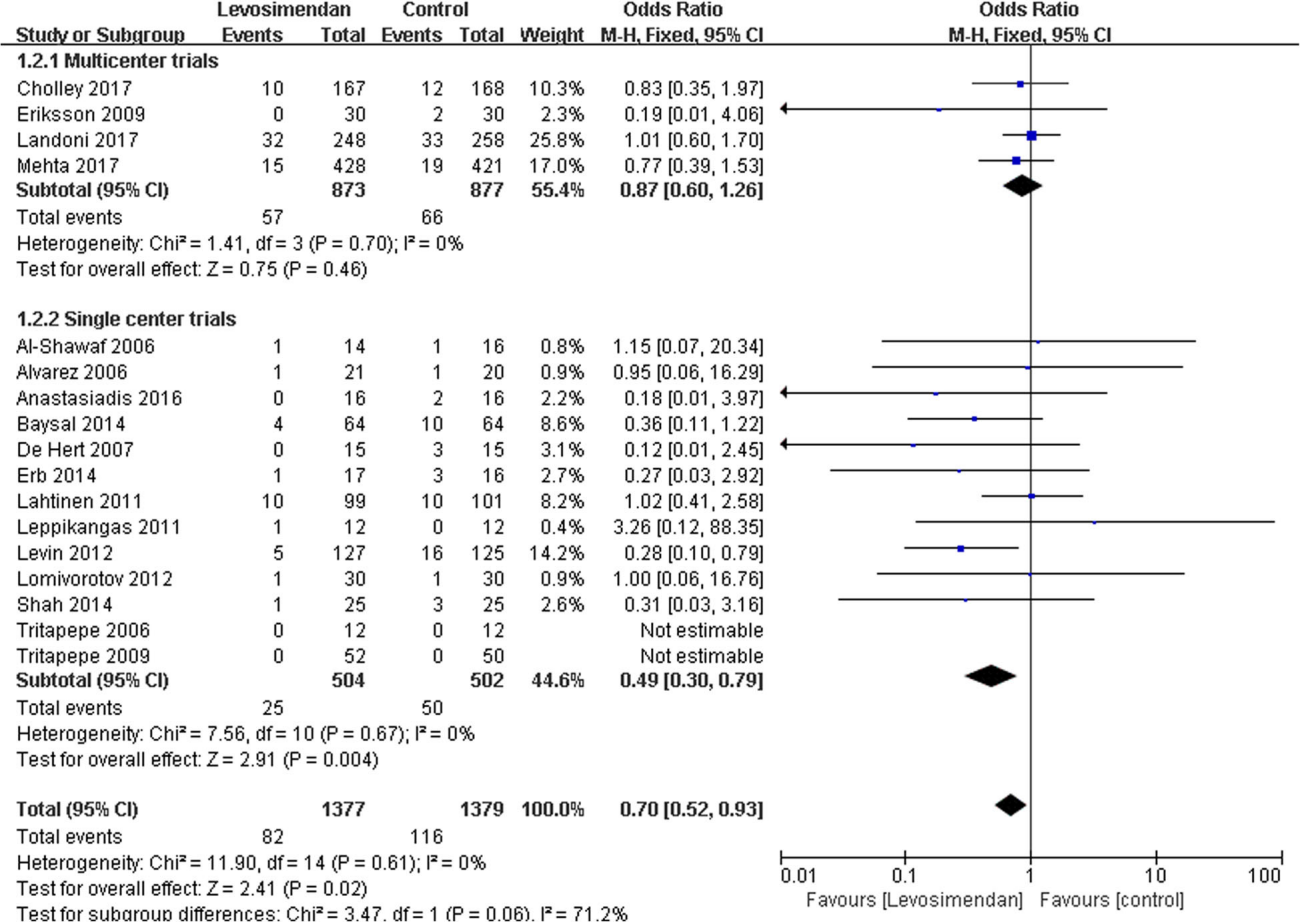
A subgroup meta-analysis of the effect of levosimendan therapy on postoperative mortality according to the single-center or multicenter nature of the trials. M-H, Mantel-Haenszel

We performed further subgroup analyses of the trials according to whether they were single-center or multicenter trials. The results showed that levosimendan therapy reduced the risk of death in single-center trial subgroups (RR 0.49; 95% CI, 0.30 to 0.79; *p* = 0.004). However, in multicenter trial subgroups, there was no significant difference in mortality between patients who received levosimendan therapy and those who did not receive levosimendan (RR 0.87; 95% CI, 0.60 to 1.26; *p* = 0.46) (Fig. 6).

### Subgroup analyses of trials based on quality

**Fig. 7:**
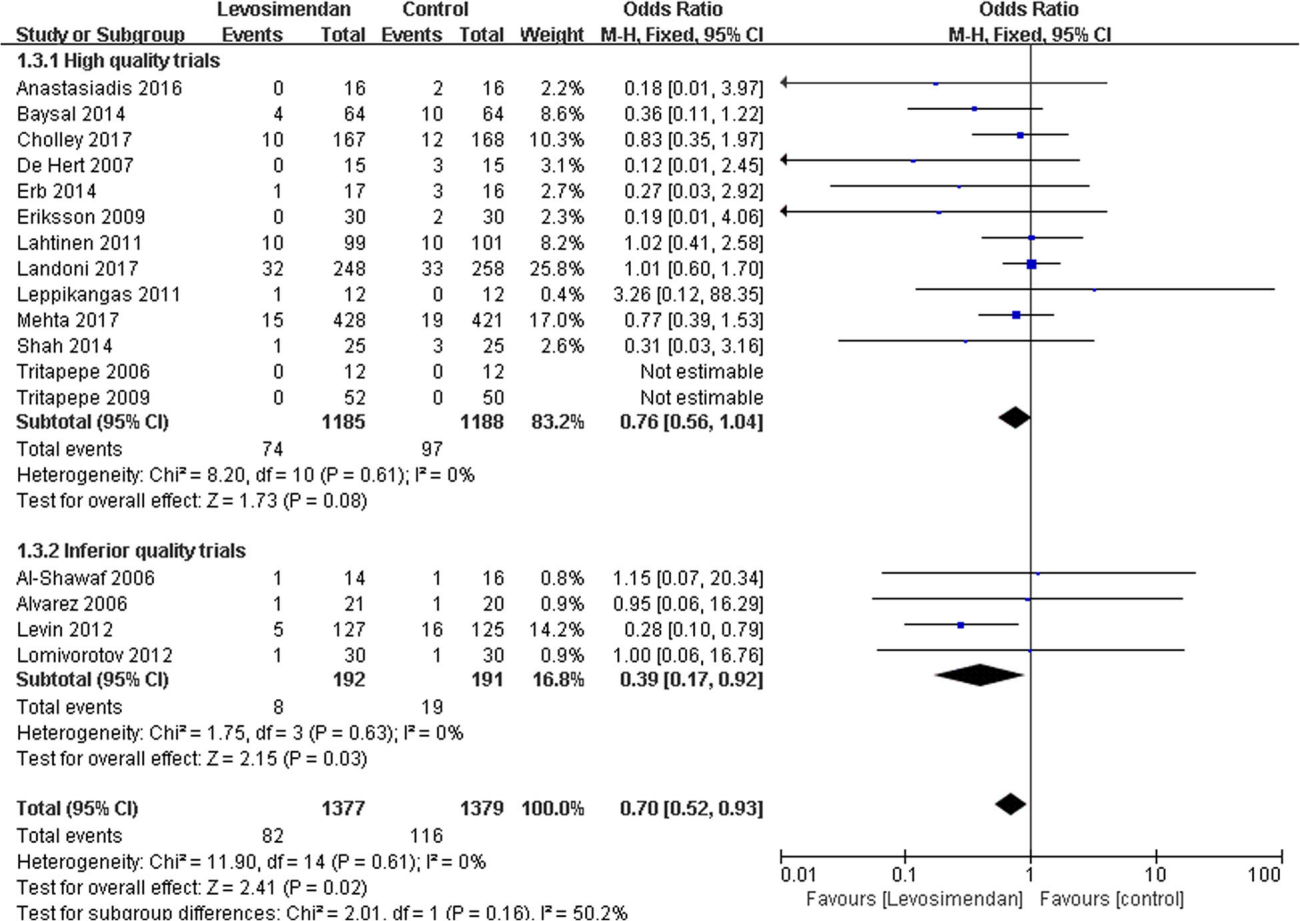
A subgroup meta-analysis of the effect of levosimendan therapy on postoperative mortality according to trial quality. M-H, Mantel-Haenszel

We analyzed the high-quality and inferior-quality trials separately. Subgroup analyses of four trials of inferior quality comparing levosimendan to controls identified association between levosimendan and mortality (RR 0.70; 95% CI, 0.52 to 0.93; *p* = 0.02). However, levosimendan therapy did not reduce the risk of death in high-quality trials (RR 0.76; 95% CI, 0.56 to 1.04; *p* = 0.08) (Fig. 7).

### Subgroup analyses of EF

**Fig. 8:**
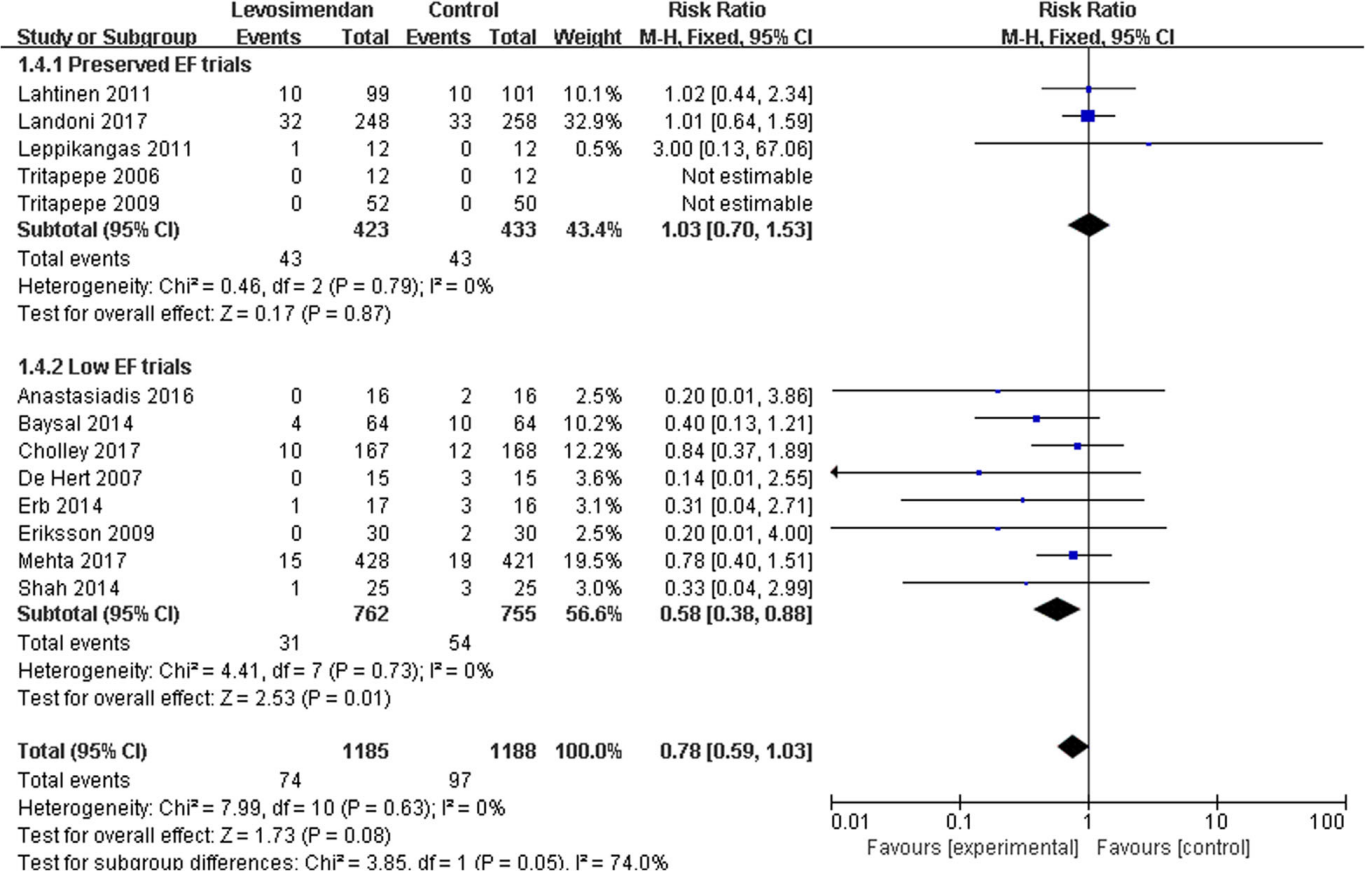
A subgroup meta-analysis of the effect of levosimendan therapy on postoperative mortality according to preoperative ejection fraction (EF). M-H, Mantel-Haenszel

This study found that the benefit of levosimendan was greatest in patients with reduced EF [5, 6]. Therefore, we performed subgroup analyses of the high-quality trials according to EF. Subgroup analysis indicated that the benefit of levosimendan was confined to the low-EF subgroup (RR 0.58; 95% CI, 0.38 to 0.88; *p* = 0.01). However, no benefit was observed in the preserved-EF subgroup (RR 1.03; 95% CI, 0.70 to 1.53; *p* = 0.87) (Fig. 8).

### Secondary outcomes

Eight studies reported the initiation of renal replacement therapy as an outcome. Overall, there was a significant reduction in the risk of renal replacement therapy with levosimendan use (RR 0.62; 95% CI, 0.45 to 0.87; *p* = 0.006). Subgroup analysis indicated that the benefit was confined to the low-EF studies (RR 0.54; 95% CI, 0.34 to 0.85; *p* = 0.007) (Additional file 1).

A total of ten studies reported ICU length of stay as an outcome. Overall, there was a significant reduction in the duration of ICU stay (MDR −13.50; 95% CI, −23.80 to −3.21; *p* = 0.01). There was a significant reduction in the preserved-EF subgroup (MDR -7.69; 95% CI, −11.23 to −4.15; *p* < 0.0001) and in the low-EF subgroup (SMD −17.19; 95% CI, −31.43 to −2.94; *p* = 0.02) (Additional file 2).

### Serious adverse events

Twelve trials (including 2592 patients) reported data on postoperative atrial fibrillation. Pooled analysis of all studies and subgroup analyses showed no significant impact on the risk of postoperative atrial fibrillation (RR 0.96; 95% CI, 0.80 to 1.15; *p* = 0.67) (Additional file 3). Postoperative hypotension was documented in five trials (1985 patients). Pooled analysis showed that levosimendan use significantly increased the incidence of hypotension (RR 1.27; 95% CI, 1.04 to 1.55; *p* = 0.02). Subgroup analysis indicated that the adverse effect was confined to the low-EF studies (RR 1.28; 95% CI, 1.02 to 1.61; *p* = 0.03) (Additional file 4).

## Discussion

Levosimendan is used to reduce mortality in patients undergoing cardiac surgery; however, recently published large-scale trials [9–11] did not provide evidence for this benefit. In this meta-analysis of randomized clinical trials of levosimendan therapy in patients undergoing cardiac surgery, the results showed that levosimendan therapy reduced the risk of death in single-center trials and in trials of inferior quality but that this benefit of levosimendan on survival was not shown in multicentric or high-quality trials. However, levosimendan therapy was associated with reduced mortality in patients with preoperative ventricular dysfunction. Furthermore, in these patients, levosimendan therapy results in less renal replacement therapy and shorter stays in the ICU.

Previous meta-analyses of small randomized trials showed a significant reduction in mortality among patients who received levosimendan treatment compared to controls among patients undergoing cardiac surgery [7, 8]. However, the benefit of levosimendan on survival was not shown in recent large-scale trials [9–11]. In our updated analysis, we included two recently published large-scale studies. Similar to a previous meta-analysis [7], our result showed that levosimendan therapy was associated with a significant reduction in mortality in patients undergoing cardiac surgery. However, subgroup analyses showed that levosimendan therapy did not reduce the risk of death in multicentric or high-quality trials. Eligible patients in many multicentric or high-quality trials had ventricular systolic dysfunction [10, 15], while patients included in the other trials had preserved left ventricular systolic function with EF exceeding 40% [8, 9, 12, 19]. Previous studies showed that levosimendan improves clinical outcomes in patients with left ventricular dysfunction [16, 25]. Patients with reduced EF may benefit more from levosimendan therapy. Therefore, we performed subgroup analyses of high-quality trials according to EF. Interestingly, patients with preoperative better left ventricular systolic function did not benefit from levosimendan therapy while levosimendan therapy was associated with a significant reduction in mortality in patients with preoperative low EF. Therefore, our meta-analysis indicates that levosimendan is only recommended for use in patients undergoing cardiac surgery with preoperative poor left ventricular function.

Postoperative AKI is a common complication in patients undergoing cardiac surgery [27]. Some studies have reported that levosimendan therapy in patients undergoing cardiac surgery is associated with lower renal replacement therapy and a shorter duration of mechanical ventilation [5, 28, 29]. However, controversial or negative results on the effect of levosimendan have been reported [18]. Our analysis showed a significant reduction in the risk of renal replacement therapy with levosimendan therapy in patients undergoing cardiac surgery. In addition, we also found that levosimendan therapy reduced mechanical ventilation duration in patients undergoing cardiac surgery. Subgroup analysis indicated that these benefits were confined to low-EF studies. Levosimendan might improve renal function in cardiac surgery due to its ability to improve cardiac systolic function and systemic hemodynamics [30]. In acute decompensated heart failure, levosimendan has an immediate renoprotective effect, which is mediated by an increase in renal blood flow resulting from selective renal arterial and venous vasodilating action [31]. One study also indicated that levosimendan therapy induced vasodilation, preferentially of preglomerular resistance vessels, thereby increasing both renal blood flow and glomerular filtration rate without jeopardizing renal oxygenation after cardiac surgery with cardiopulmonary bypass [32].

The improvement in cardiac systolic function induced by levosimendan is due to two mechanisms – vasodilation and increased contractility [33]. In this meta-analysis, postoperative hypotension was documented in four trials (1600 patients). Pooled analysis showed no association between levosimendan and postoperative hypotension. A possible reason for this may be that the reduction in systemic vascular resistance is compensated for by an increased cardiac index. Since most of these hypotension episodes often occurred following the administration of a loading dose, hypotension may be avoided by eliminating the loading dose while the favorable hemodynamic effects of levosimendan can be obtained through continuous infusion [23, 34].

With regard to other adverse effects, postoperative arterial fibrillation was also observed. Our analysis showed that levosimendan therapy did not increase the incidence of postoperative arterial fibrillation. However, levosimendan use significantly increased the incidence of hypotension in patients with preoperative ventricular systolic dysfunction. Although levosimendan can be considered to be a well-tolerated agent that can provide an important treatment option for cardiac systolic dysfunction following cardiac surgery [35, 36], levosimendan should be used with caution in patients with hemodynamic instability.

There were several limitations in this meta-analysis. Levosimendan dosing and drug-delivery methods varied between the trials. Four studies used an infusion without a bolus, and two studies used bolus dosing without an infusion. Additionally, we performed a subgroup analysis according to the mean EF. As a result, some studies [9, 18, 19] that were classified as preserved-EF studies included some patients with EF ≦40%. Furthermore, we were unable to access individual patient data. Therefore, some means and standard deviations of the patients’ ICU length of stay data were estimated according to the method described by Hozo [14].

## Conclusion

In summary, the available evidence from our updated meta-analysis suggests that levosimendan therapy reduced the risk of death in single-center trials and in trials of inferior quality, but there was no benefit of levosimendan on survival in multicentric and in high-quality trials. However, levosimendan therapy was associated with reduced mortality in patients with preoperative ventricular systolic dysfunction. Furthermore, in these patients, levosimendan therapy resulted in less renal replacement therapy and shorter ICU stays. However, patients with normal left ventricular systolic function cannot benefit from levosimendan therapy. Additionally, levosimendan should be used with caution in patients with hemodynamic instability because levosimendan use significantly increased the incidence of hypotension in patients with preoperative ventricular systolic dysfunction who were undergoing cardiac surgery.

## Additional files

Additional file 1: The effect of levosimendan on postoperative renal replacement therapy in patients undergoing cardiac surgery. (PNG 11 kb)

Additional file 2: The effect of levosimendan on duration of ICU stay in patients undergoing cardiac surgery. (PNG 13 kb)

Additional file 3: The effect of levosimendan on postoperative atrial fibrillation in patients undergoing cardiac surgery. (PNG 13 kb)

Additional file 4: The effect of levosimendan on postoperative hypotension in patients undergoing cardiac surgery. (PNG 9 kb)

## Abbreviations

CABG: Coronary artery bypass grafting
EF: Ejection fraction
EF: Ejection fraction
HVR: Heart valve replacement
IABP: Intra-aortic balloon counterpulsation
ICU: Intensive care unit
LCOS: Low cardiac output syndrome
MDR: Mean Difference Risk
SMD: Standard Mean Difference
TSA: Trial sequential analysis
USA: United States of America
