# Supplementary figures and images for: Effect of levosimendan on prognosis in adult patients undergoing cardiac surgery: a meta-analysis of randomized controlled trials
Source: Crit Care. 2017 Oct 17;21:253. doi: 10.1186/s13054-017-1848-1 (PMC5645931; doi:10.1186/s13054-017-1848-1)

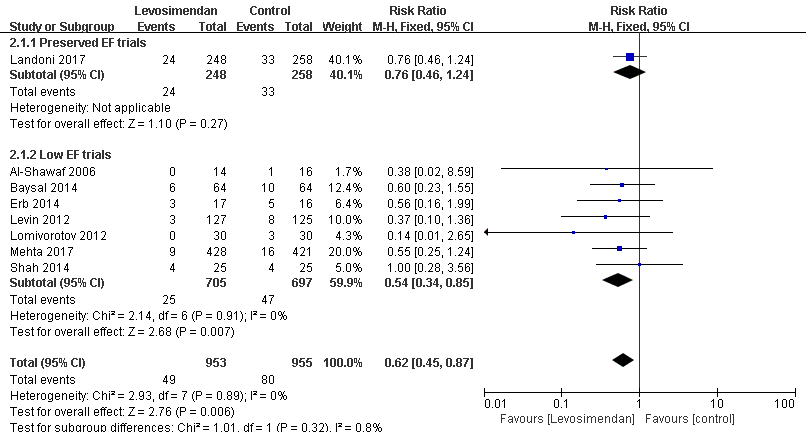

Supplement: Supplementary file 1 — The effect of levosimendan on postoperative renal replacement therapy in patients undergoing cardiac surgery. (PNG 11 kb) [file 13054_2017_1848_MOESM1_ESM.png]

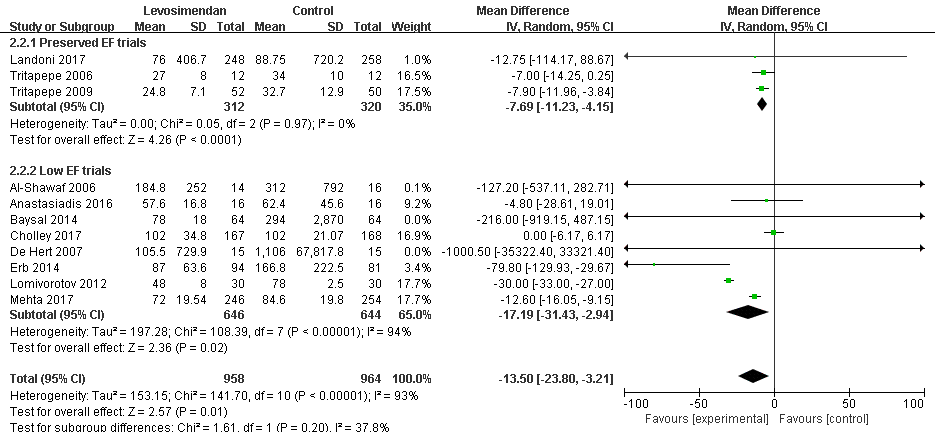

Supplement: Supplementary file 2 — The effect of levosimendan on duration of ICU stay in patients undergoing cardiac surgery. (PNG 13 kb) [file 13054_2017_1848_MOESM2_ESM.png]

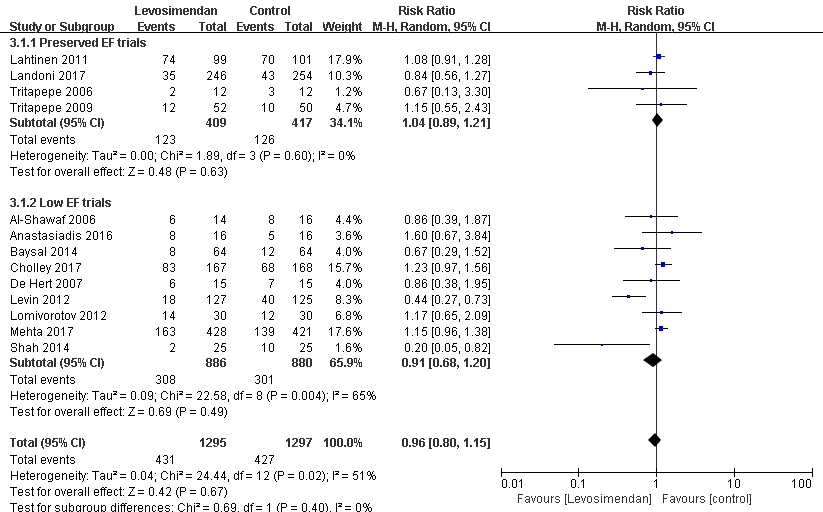

Supplement: Supplementary file 3 — The effect of levosimendan on postoperative atrial fibrillation in patients undergoing cardiac surgery. (PNG 13 kb) [file 13054_2017_1848_MOESM3_ESM.png]

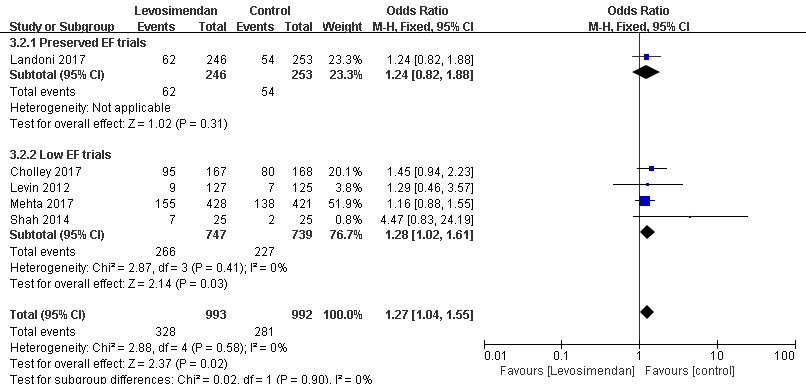

Supplement: Supplementary file 4 — The effect of levosimendan on postoperative hypotension in patients undergoing cardiac surgery. (PNG 9 kb) [file 13054_2017_1848_MOESM4_ESM.png]
